# Antibodies to Senescent Antigen and C3 Are Not Required for Normal Red Blood Cell Lifespan in a Murine Model

**DOI:** 10.3389/fimmu.2017.01425

**Published:** 2017-10-30

**Authors:** Krystalyn E. Hudson, Karen de Wolski, Linda M. Kapp, Amanda L. Richards, Matthew J. Schniederjan, James C. Zimring

**Affiliations:** ^1^Bloodworks Northwest Research Institute, Seattle, WA, United States; ^2^Department of Pathology and Laboratory Medicine, Emory University, Atlanta, GA, United States; ^3^Department of Laboratory Medicine, Division of Hematology, University of Washington, Seattle, WA, United States; ^4^Department of Internal Medicine, Division of Hematology, University of Washington, Seattle, WA, United States

**Keywords:** red blood cell clearance, complement C3, red blood cell lifespan, antibodies, senescent antigen, naturally occurring antibodies

## Abstract

Red blood cells (RBCs) have a well-defined lifespan, indicating a mechanism by which senescent cells of a certain age are removed from circulation. However, the specifics by which senescent cells are recognized and removed are poorly understood. There are multiple competing hypotheses for this process, perhaps the most commonly cited is that senescent RBCs expose neoantigens [or senescent antigen(s)] that are then recognized by naturally occurring antibodies, which opsonize the senescent cells and result in clearance from circulation. While there are a large volume of published data to indicate that older RBCs accumulate increased levels of antibody on their surface, to the best of our knowledge, the causal role of such antibodies in clearance has not been rigorously assessed. In the current report, we demonstrate that RBC lifespan and clearance patterns are not altered in mice deficient in antibodies, in C3 protein, or missing both. These data demonstrate that neither antibody nor C3 is required for clearance of senescent RBCs, and questions if they are even involved, in a murine model of RBC lifespan.

## Introduction

Red blood cells (RBCs) have a tightly regulated circulatory lifespan. The precise modifications that senescent RBCs undergo, which leads to their removal from circulation, remain incompletely understood. Previous studies in both humans and mice implicate similar pathways lead to senescent RBC clearance, including modulation of CD47 and phosphatidylserine expression, changes in density, and complement C3 deposition (1–8). However, among the most widely accepted explanations, is that age-related changes on the RBC surface expose neo-epitopes (broadly termed “senescent antigen”) that are recognized by naturally occurring antibodies, which then opsonize the senescent RBCs and lead to clearance. Indeed, Kay first reported that as RBCs age, more IgG is detectable on the RBC surface (9, 10), and multiple investigators have reproduced these findings over the past few decades. There have been several different hypotheses regarding the molecular basis for senescent antigen, including alteration and/or clustering of Band 3, alterations of surface carbohydrates and/or proteolysis of other proteins, and accumulation of oxidized and denatured proteins (11–17). Moreover, Lutz and colleagues reported that efficient phagocytosis of oxidized RBCs required C3b deposition in addition to Band 3 clustering and presence of anti-Band 3 autoantibodies (18, 19), suggesting a coordinated response between age-related changes in RBCs and the innate immune system. Thus, the current model is that senescent antigen accumulates with RBC age, naturally occurring autoantibodies bind, prompt complement deposition and lead to subsequent clearance of older RBCs.

Despite the panoply of data and the multitude of papers that have been published on the role(s) of naturally occurring autoantibodies and complement in older RBC clearance through binding senescent antigen, to the best of our knowledge, the requirement of such autoantibodies has not been rigorously tested. The principal data claiming to demonstrate that senescent antigen-specific “Immunoglobulin (Ig) is required for phagocytosis” is based upon *in vitro* studies in which senescent RBCs are incubated with IgG followed by measurement of *in vitro* consumption by phagocytes (9). Additional data generated with reinfusion of *ex vivo* biotin-labeled RBCs correlated accumulation of IgG with RBC removal (20). However, in addition to problems extrapolating from *in vitro* to *in vivo* biology, assessing whether RBC-labeling techniques inadvertently damage RBCs before reinfusion, the previous studies can only assess whether an entity is capable of causing phagocytosis *in vitro* or is associated with clearance *in vivo* but lack the rigor to test whether it is required. Herein, we utilize transgenic mouse models with selective deletions in Ig and C3 genes to directly test the requirement of antibodies and complement in clearance of senescent RBCs. We report that neither Ig nor C3 are required for RBC clearance. Moreover, we demonstrate that erythrophagocytosis by splenic leukocytes is not altered in the absence of antibodies or complement. Together, these findings demonstrate that neither antibodies nor complement C3 are required for clearance of senescent RBCs in a murine system.

## Materials and Methods

### Mice

C57BL/6 (B6), UBI-GFP [C56BL/6-Tg(UBC-GFP)30Scha/J, stock #004353], Jh (B6.129P2-Igh-J^tm1Cgn^/J, stock #002438), and C3KO (B6;129S4-C3^tm1/Crr^/J, stock #003641) were purchased from Jackson laboratories. UBI-GFP mice express green fluorescent protein (GFP) under the ubiquitin C promoter and have detectable GFP expression in every tissue examined (21). Jh mice lack Jh genes and are B cell deficient; as such, Jh mice have no detectable IgM or IgG (22). C3 null mice (C3KO) lack the complement component 3, which is essential for activation of the complement cascade and innate immune responses (23). All breeding was performed in the BloodworksNW vivarium. The UBI-GFP, Jh, and C3KO mice are each on a C57BL/6 background. Breeding was carried out to generate UBI-GFP heterozygotes on backgrounds of Jh, C3KO, or double knockout mice (JhxC3KO). All experiments were performed according to approved Institutional Animal Care and Use Committee (IACUC) procedures.

### Kinetics and Duration of RBC Lifespan

Red blood cell lifespan was determined by enumerating donor RBCs by flow cytometry of GFP^+^ RBCs, as previously described (24). UBI-GFP RBCs were harvested from each of 4 UBI-GFP donor strains and 100 μL of packed RBCs were transfused into the corresponding recipient strain of the same genetic background, but GFP negative.

### *In Vivo* Erythrophagocytosis

The assessment of cellular consumption of RBCs was carried out as previously described in methodological detail (25).

### Statistical Analysis and Graphing

Statistical significance for three or more groups was evaluated by a one-way ANOVA followed by a Bonferroni’s posttest. Significance was set at *p* ≤ 0.05 and *≤0.05, **≤0.01, ***≤0.001. Flow plots were generated using FlowJo software, and graphs were generated using GraphPad Prism.

## Results

**Figure 1:**
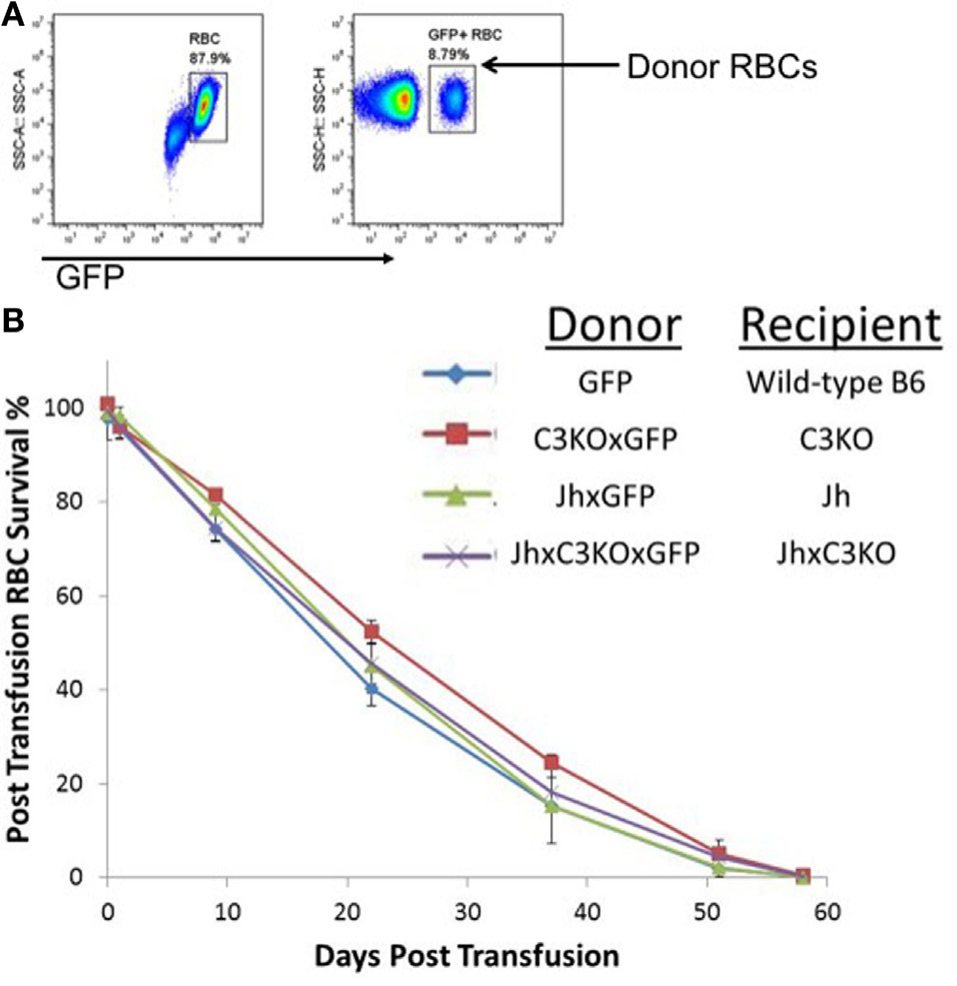
Red blood cell (RBC) clearance is normal in mice deficient in either immunoglobulin, C3, or both. RBCs from GFP^+^ mice of the indicated strains were transfused into GFP^−^ mice of the same strains. **(A)** Transfused RBCs were visualized by flow cytometry after gating on the RBC population by forward and size scatter (representative panels shown). **(B)** Percentage of circulating RBCs was determined at each of the indicated time points, longitudinally in the same mice for the length of the experiment. To allow monitoring of RBCs for the entire RBC lifespan, 100 μL of GFP^+^ RBCs were transfused, resulting in an approximate 10% of RBCs at the beginning of the experiment [representative flow cytometry shown in panel **(A)**]. There were three mice included in each group; error bars represent SD. A representative experiment is shown; similar results were observed in three out of three repetitions.

To test the requirements of antibody and/or complement C3 for the clearance of RBCs as they senesce, the UBI-GFP transgene was bred onto Jh, C3KO, or JhxC3KO backgrounds. GFP^+^ RBCs were transfused into GFP^−^ recipients, and lifespan of the RBCs was determined by enumerating GFP^+^ RBCs in recipient circulation over time by longitudinal analysis of individual animals, as described in Ref. (24). No significant difference was observed in RBC lifespan or clearance kinetics between wild-type B6, Jh, C3KO, or JhxC3KO mice (Figure 1). The use of GFP as an intracellular marker removes the requirement to label RBCs using dyes or tracers, and thus obviates concerns around damaging the RBCs during labeling and/or modifying surface antigens as a result of covalent attachment of label. This approach also eliminates concerns around introduction of C3 or Ig by donor mice, as donors and recipients both lack the same gene products. Moreover, given that each recipient received a syngeneic transfusion and RBC clearance was comparable between all strains (including control B6), these data demonstrate that the nature of the transgenic mouse did not affect RBC clearance pathways. In addition, these findings indicate that neither C3 nor Ig is required for senescent RBCs to be cleared at the end of their normal lifespan.

**Figure 2:**
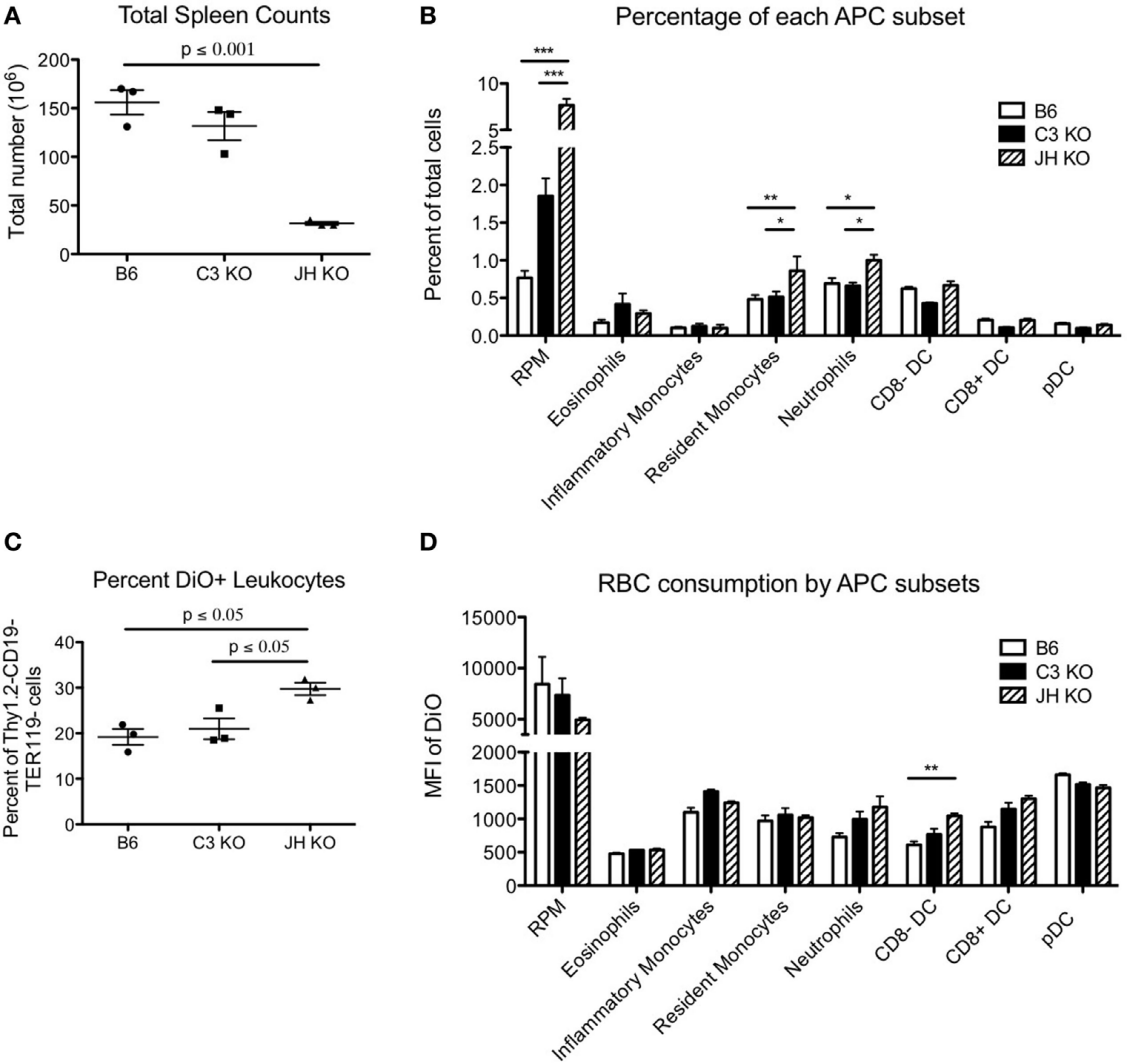
Consumption of red blood cells (RBCs) by different phagocytic subsets. RBCs were labeled with DiO^+^ and transfused into the indicated recipient strains. Subsets of recipient splenocytes were enumerated by flow cytometry using standard staining panels for lineage specific antigens **(A,B)**. The extent of RBC consumption was assessed by gating on each indicated lineage and assessing DiO fluorescence, which reflects the extent of RBC consumption (8) **(C,D)**. Only events that stained negative with anti-TER119 (an RBC specific antigen in mice) were counted, so as to avoid evaluating phagocytes with RBCs stuck to the surface, as opposed to consumed. Representative data are shown for one experiment with three mice per group. Similar results were obtained in three out of three repetitions. For cellular subset analysis, T cells, B cells, and RBCs were excluded from total live leukocytes by gating on Thy1.2^−^CD19^−^TER119^−^ cells. Red pulp macrophages (RPM) are defined as CD11c^int^F480^+^CD11b^lo/−^, CD8a^−^ dendritic cells (DCs) express CD11c^hi^CD11b^+^CD8a^−^ whereas CD8a^+^ DCs are CD11c^hi^CD11b^−^CD8a^+^, and plasmacytoid DCs (pDCs) are CD11c^int^Ly6C^hiL^Ly6G^−^B220^+^. CD11c^int/−^CD11b^+^Ly6G^−^Ly6C^hi^CD115^+^ inflammatory monocytes, CD11c^int/−^CD11b^+^Ly6G^−^Ly6C^hi^CD115^−^ resident monocytes, CD11c^int/−^CD11b^+^Ly6G^+^ neutrophils, and CD11c^int/−^CD11b^+^Ly6G^−^ with high side scatter eosinophils were also be delineated.

While the kinetics of RBC clearance was not significantly different in the absence of antibody and/or C3, this does not necessarily indicate similarities between strains in how the reticuloendothelial system removes senescent cells, as recognition and binding of naturally occurring antibodies and complement components may divert RBCs to specific leukocytes. To further investigate this issue, we analyzed the distribution of RBC consumption by leukocytes. The absence of B cells in Jh mice resulted in a significant decrease in overall numbers of splenocytes (Figure 2A). However, there was a significant fourfold increase in the percentage of red pulp macrophages (RPM), and also an increase (albeit less pronounced) in resident monocytes and neutrophils (Figure 2B). By contrast, there was no significant difference in percentages of different dendritic cell (DC) subsets, eosinophils, or inflammatory monocytes. C3KO had similar total splenocyte counts as B6 mice, with slight decreases in percentages of DC subsets and a small increase in RPM. Overall, the percentage of DiO^+^ leukocytes (white cells that had consumed RBCs) was highest in Jh mice, followed by C3KO, and lowest in B6 mice (Figure 2C). Measuring the MFI of a given populations as an indication of the amount of RBCs consumed per cell indicated that Jh mice had a significant increase in consumption by CD8^−^ DCs and decreased consumption by RPM (Figure 2D). No other statistically significant differences were observed; no differences were detected between C3 and B6 mice.

## Discussion

In aggregate, these data demonstrate that in the absence of antibodies, C3, or both simultaneously, circulatory lifespan of RBCs is not altered. While the amount of RBCs consumed by any particular macrophage is slightly lower in Jh mice, this is offset by the increased number of macrophages present, such that overall consumption is not altered. Together, the findings in this paper bring into serious question the hypothesis that normal RBC senescence is mediated by Igs and/or requires complement C3.

This manuscript is, to the best of our knowledge, the first paper to investigate RBC survival in the absence of both Ig and C3. However, there has been a report of RBC lifespan juxtaposing normal BALB/c mice with agammaglobulinemic mice due to severe combined immunodeficiency (SCID) due to a mutation in Prkdc^scid^ (BALB/c SCID) (26). The aim of this paper was a mechanistic evaluation of the role that antibodies play in the premature removal of neuraminidase treated RBCs, which have greatly accelerated clearance (4-day lifespan vs. 55 days for untreated cells). Neuraminidase treated RBCs cleared in 3 days in wild-type mice and 4 days in SCID mice. Untreated RBCs were used as a control, with no difference observed between wild-type and SCID mice, although little clearance was observed for untreated cells as the experimental observation was limited to 4 days. Rather than use these data to reject the hypothesis that antibodies were required, the authors interpreted these data as an indication that antibodies were indeed involved, but that there must be “an alternative pathway that is antibody-independent.” This conclusion seems to hinge on the observation that deletion of antibodies slightly delayed clearance by 1 day for neuraminidase treated cells (although the significance of this is unclear as no statistical analysis was provided). Although using “agammaglobulinemic mice” was a very reasonable approach, an additional problem is that the SCID mutation leaks on certain genetic backgrounds, including BALB/c, resulting in detectable serum Igs (27).

The current report uses Jh mice, which have a deletion of an essential part of the Ig gene, decreasing (if not eliminating) the risk of leakiness. We observed no difference in RBC lifespan in agammaglobulinemic mice vs. wild-type controls. Likewise, we observed no difference in RBC lifespan in C3KO mice. C3b, a product of C3, has been argued to be essential to antibody-mediated clearance of senescent RBCs (18, 28, 29). C3 has also been argued to be capable of mediating RBC clearance in the absence of antibody, at least in the context of clearance of stored RBCs (30, 31). If Igs or C3 were required for RBC clearance, then the removal of either (or both) would be predicted to result in a longer RBC circulatory lifespan; such was not observed. While it is not logically feasible to exclude the possibility of any redundant pathway, the normal RBC lifespan in the indicated strains would require the supposition of a third pathway to rescue the hypothesis, one that maintained the same pattern of clearance by reticuloendothelial cells. This is not to imply, in any way, that a very strong association does not exist between aging RBCs and surface Ig and/or C3; such observations have been made by multiple investigators over decades, and are not in dispute. However, the current data do undermine the notion that association is a causal relationship and provide evidence that neither surface Igs or C3 (or both) are required for clearance of senescent RBCs. Finally, we cannot rule out that compensatory RBC clearance mechanisms evolved due to an absence of antibodies, complement C3, or both in response to their genetic modifications. However, we think this is unlikely due to the similarity of RBC clearance and consumption patterns in the different transgenic mice compared with controls.

Many hypotheses have been put forth to explain the mechanism(s) of clearance of senescent RBCs but much controversy remains. Much work has been done on human RBCs, giving rise to correlations (e.g., increased Ig on RBCs over time). However, causality can only be specifically tested by removing a variable and observing its effect. Typically, this is neither logistically nor ethically feasible in humans, and is a limitation of experimentation on human systems. The plethora of genetically modified mice allow for removal of a specific potential maker (or mechanism) of senescent RBCs with relative ease. In addition, human studies have not been able to directly test this hypothesis *in vivo* in a causal way, and has had to use *in vitro* modeling. For example, much of the human data have had to resort to using density centrifugation to separate “young” and “old” RBCs to identify markers of senescence, and recent data have demonstrated that these cell populations are not pure. Alternatively, much human data have used heat or chemically damaged RBCs to model aging, and it is unclear that such models reflect the natural aging process. Moreover, the murine model provides a powerful platform, such as the exploitation of GFP^+^ mice, that avoids the need for excessive *ex vivo* manipulations (e.g., biotinylation) before reinfusion. As such, animal modeling provides an opportunity to test causality; but in doing so, raises the possibility that the biology of the animal may not reflect that of humans. Mice are not humans, and as such, the data herein may not reflect human biology. That having been said, murine and human RBC biology lines up in many ways, most importantly to the current studies, that Ig accumulates on RBCs as they age in both species. As such, the current studies provide new evidence arguing against an essential role of Ig or C3 for RBC clearance in mice, and suggest the same may be true in humans. Should a way to ethically test this directly in humans and *in vivo* be devised, then extending the direct scrutiny of this hypotheses in humans will be an important next step.

## Ethics Statement

All procedures were performed according to approved BloodworksNW Institutional Animal Care and Use Committee (IACUC) protocols.

## Author Contributions

KH, Kd, LK, AR, MS, and JZ each performed experiments and analyzed data contained in this work. KH and JZ authored the manuscript and Kd, LK, AR, and MS provided revisions. All the authors read and approved of the manuscript.

## Conflict of Interest Statement

KH, Kd, LK, AR, and MS have no conflicts of interest to declare. JZ serves on the Scientific Advisory Board for Rubius Therapeutics and consults or Surface Oncology and Sinopia Biosciences.
